# Brentuximab Vedotin Infusion Reaction Management: A Case Study

**Published:** 2017-09-01

**Authors:** Holly Comer, Kimbra Cardwell

**Affiliations:** Washington University School of Medicine, Siteman Cancer Center, St. Louis, Missouri

## Abstract

We report a case of a grade 3 (Common Terminology Criteria for Adverse Events [CTCAE]) infusion reaction to brentuximab vedotin (Adcetris), in a patient with refractory Hodgkin lymphoma, at a large National Cancer Institute–designated cancer center in the Midwest (National Cancer Institute, 2010). Acute infusion reaction management and subsequent premedication strategies are outlined.

Ms. R is a 30-year-old woman who presented with stage IV Hodgkin lymphoma at the age of 29. Initial staging revealed lymphadenopathy above and below the diaphragm, as well as fluorodeoxyglucose (FDG)-avid lung lesions, splenic lesions, and multiple sites of bony involvement. Bone marrow biopsy was negative.

She was treated with six cycles of chemotherapy with doxorubicin, bleomycin, vinblastine, and dacarbazine (ABVD), to which she obtained a complete response by positron emission tomography–computed tomography (PET-CT) criteria. Ten months after chemotherapy completion, she presented with new PET-avid adenopathy in the cervical and paratracheal regions, and a biopsy revealed recurrent Hodgkin lymphoma. Salvage chemotherapy was administered with ifosfamide carboplatin, and etoposide (ICE). After two cycles of salvage chemotherapy, a PET-CT confirmed a complete response, and she proceeded to an autologous stem cell transplant with a preparative regimen of carmustine, etoposide, cytosine arabinoside, and melphalan (BEAM).

Brentuximab vedotin consolidation therapy was prescribed in the post-transplant consolidation setting, beginning 45 days after stem cell reinfusion, given the patient’s high risk for recurrence. This strategy was based upon the results of the AETHERA phase III clinical trial (Moskowitz et al., 2015), showing improvement in progression-free survival with brentuximab vedotin consolidation therapy, post autologous transplant.

The first dose of brentuximab vedotin was administered without difficulty, at full dose (1.8 mg/kg) at a standard infusion time of 30 minutes. The second dose of brentuximab vedotin was complicated by nausea, chest pain, and dysphagia within 10 minutes of medication initiation. Upon the emergence of these symptoms, the brentuximab vedotin infusion was held. Vital signs were stable, with a temperature of 36.9˚C, pulse 84, respirations of 20, and blood pressure of 107/67 mm Hg. Oxygen saturations were 99% on room air. Diphenhydramine (50 mg) was administered intravenously (IV), along with 20 mg of IV famotidine. An electrocardiogram (ECG) was obtained, which was unremarkable, showing normal sinus rhythm. Fifteen minutes later, the symptoms of chest pain and shortness of breath persisted, so hydrocortisone at 100 mg IV was administered, with an additional 25 mg of IV diphenhydramine and 20 mg of IV famotidine. Intraveous granisetron was given for nausea. Thirty minutes after onset, the chest pain was persistent, and oxygen saturations were normal. Hydrocortisone (50 mg) was administered intravenously, and Ms. R’s condition improved, with resolution of her symptoms within 30 minutes of the second hydrocortisone dose.

The brentuximab vedotin was restarted 30 minutes after symptom resolution at a decreased infusion rate to be administered over 60 minutes. Thirty minutes later, however, Ms. R developed tingling and numbness in her feet and tongue. The brentuximab vedotin infusion was again held, and 100 mg of IV methylprednisolone was administered. Ms. R’s symptoms resolved within 40 minutes, and the brentuximab vedotin infusion was able to be continued over a prolonged period of more than 4 hours. Vital signs were checked every 15 minutes during the infusion reaction and remained stable throughout. The infusion was discontinued with 40 mg of drug remaining, due to the prolonged infusion time.

Given the clear benefits of brentuximab consolidation in improving progression-free survival post transplant (Moskowitz et al., 2015) in high-risk Hodgkin lymphoma, it was thought the benefit of brentuximab vedotin consolidation outweighed the possible risks of subsequent infusions.

Upon reviewing the available literature regarding brentuximab vedotin hypersensitivity reactions, which will be outlined in the discussion summary, we instituted the premedication strategy for subsequent infusions outlined in the Table on p 628.

Standard epinephrine and methylprednisolone were available at the bedside in the event of any anaphylactic reaction. This regimen was chosen based on the clinical rationale for H1 and H2 blockade, as well as corticosteroid and antipyretic coverage, in the prevention of hypersensitivity reactions, not classified as anaphylaxis. With the institution of the outlined premedications, Ms. R tolerated subsequent infusions well, at full dose and at standard infusion rates, with no documented infusion reactions, and was able to complete a total of 16 cycles of consolidation therapy.

Infusion reactions have been reported with brentuximab vedotin therapy in both clinical trial settings (Younes et al., 2010) and in the post clinical trial experience (Baxley, Kumm, Bishop, Medina, & Holter-Chakrabarty, 2013). Infusion reactions can range from minor grade 1 infusion reactions, to anaphylactic reactions, requiring drug discontinuation. 

Arora, Bhatt, Liewer, Armitage, and Bociek (2015), reported a case of successful brentuximab vedotin desensitization utilizing a 12-step desensitization protocol for anaphylactic reactions, with increasing dosage and rate over 3 subsequent infusions. Similarly, DeVita, Evens, Rosen, Greenberger, and Petrich (2014) reported a successful 13-step desensitization protocol for the treatment of anaphylaxis related to brentuximab vedotin, which was unresponsive to steroid, antipyretic, antihistamine, and H2 blockade premedications.

As our patient Ms. R had a grade 2 infusion reaction, but no anaphylaxis, we utilized a strategy of steroid, antipyretic, antihistamine, and H2 blockade premedications, without deployment of the full desensitization strategy as described by Arora et al. (2015) and DeVita et al. (2014). This strategy has permitted us to continue full-dose consolidation treatment with brentuximab vedotin without any subsequent infusion reactions. We believe this strategy will provide a useful tool for clinicians in the management of grade 2/3 infusion reactions.

**Table:**
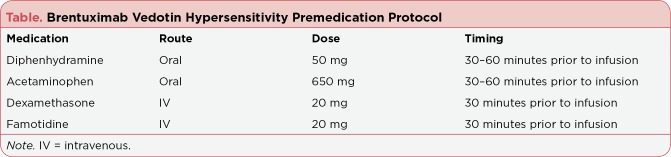
Brentuximab Vedotin Hypersensitivity Premedication Protocol

## BACKGROUND

Antibody-drug conjugates (ADCs) are one of the newer types of chemotherapy agents. Brentuximab vedotin is an ADC consisting of a monoclonal antibody targeting CD30, which is overexpressed in Hodgkin lymphoma, and anaplastic large cell lymphoma, conjugated with monomethyl auristatin E (MMAE). This microtubule-disrupting agent (MMAE) causes cell death.

The drug was developed with the help of a technology lesson from the Indian Ocean sea hare, *Dolabella auricularia* (Bouchard et al., 2014). This sea animal harbors toxic substances that protect it from being eaten. From studying these toxic substances, there arose a drug class known as the auristatins. These auristatins are conjugated with monoclonal antibodies to make the ADC brentuximab. The ADC brentuximab attaches to the tumor-associated antigen, where it is internalized into the cell. Brentuximab then releases its cytotoxic agent, which causes cell death by mitotic disruption similar to the vinca alkaloids.

Brentuximab vedotin was first approved by the US Food and Drug Administration (FDA) for the treatment of Hodgkin lymphoma and anaplastic large cell lymphoma on August 19, 2011 (Senter & Sievers, 2012). It is approved for second-line therapy in Hodgkin lymphoma and anaplastic large cell lymphoma and as consolidation post autologous transplant in Hodgkin lymphoma (Moskowitz et al., 2015).

## INFUSION REACTIONS

Brentuximab vedotin, like many monoclonal antibodies, can produce infusion reactions. In phase I clinical trials, there were two cases of anaphylaxis reported. In phase II clinical trials, 12% of patients reported infusion-related reactions (Singh, Singh, & Bhoria, 2014). 

There are two mechanisms thought to produce infusion-related reactions: anaphylaxis and anaphylactoid reactions. Anaphylaxis is a systemic reaction from the interaction between immunoglobulin E (IgE) and mast cells. Anaphylactoid reactions are cytokine-mediated, rather than IgE-mediated. Clinical manifestations tend to be similar in both types of reactions (Singh et al., 2014). The reactions most common with brentuximab vedotin are anaphylactoid, including chills, nausea, dyspnea, pruritus, pyrexia, and cough.

Patients in clinical trials for brentuximab vedotin were tested every 3 weeks for antibodies using a sensitive immunoassay. A total of 7% of patients developed positive antibodies, and 30% developed transiently positive antibodies. A higher incidence of infusion reactions were noted in these persistently positive patients. Patients were also noted to develop these reactions during the second dose, possibly allowing these antibodies to develop after the initial exposure to the medication. There are case reports of patients developing problems as far out as cycle 4.

Another interesting observation is that several of these reactions occurred in patients after transplant (DeVita et al., 2014). In the case study presented here, Ms. R was not tested for brentuximab antibodies, as she responded to conservative premedication strategies.

## DESENSITIZATION PROTOCOLS

There are several desensitization protocols available for patients who develop anaphylaxis to brentuximab vedotin. A standard 12-step protocol has been successfully used in several patients. The standard 12-step protocol may be used in severe reactions, but only a small subset of patients would require this intense method (Arora et al., 2015).

Another example of a rapid desensitization protocol involves premedication with prednisone at 50 mg at 24, 12, and 0 hours prior to infusion, followed by premedication with IV diphenhydramine, IV ranitidine, IV methylprednisolone (125 mg) and oral montelukast 30 minutes prior to the infusion (Arora et al., 2015). Another possible strategy was outlined earlier using oral diphenhydramine, oral acetaminophen, IV dexamethasone, and IV famotidine 30 minutes prior to the infusion. Using these various strategies, we can safely infuse brentuximab vedotin to patients with few reactions. The choice of regimen can be made based on the severity of reaction, specifically to the patient.

## References

[1] Arora Anubha, Bhatt Vijaya Raj, Liewer Susanne, Armitage James O, Bociek R Gregory (2015). Brentuximab vedotin desensitization in a patient with refractory Hodgkin's lymphoma.. *European journal of haematology*.

[2] Baxley Allison A, Kumm Debra E, Bishop Courtney B, Medina Patrick J, Holter-Chakrabarty Jennifer (2013). Severe infusion reactions to brentuximab vedotin in two patients with Hodgkin lymphoma previously treated with allogeneic stem cell transplantation.. *Journal of oncology pharmacy practice : official publication of the International Society of Oncology Pharmacy Practitioners*.

[3] Bouchard Hervé, Viskov Christian, Garcia-Echeverria Carlos (2014). Antibody-drug conjugates—a new wave of cancer drugs.. *Bioorganic & medicinal chemistry letters*.

[4] DeVita Michael D, Evens Andrew M, Rosen Steven T, Greenberger Paul A, Petrich Adam M (2014). Multiple successful desensitizations to brentuximab vedotin: a case report and literature review.. *Journal of the National Comprehensive Cancer Network : JNCCN*.

[5] Moskowitz Craig H, Nademanee Auayporn, Masszi Tamas, Agura Edward, Holowiecki Jerzy, Abidi Muneer H, Chen Andy I, Stiff Patrick, Gianni Alessandro M, Carella Angelo, Osmanov Dzhelil, Bachanova Veronika, Sweetenham John, Sureda Anna, Huebner Dirk, Sievers Eric L, Chi Andy, Larsen Emily K, Hunder Naomi N, Walewski Jan (2015). Brentuximab vedotin as consolidation therapy after autologous stem-cell transplantation in patients with Hodgkin's lymphoma at risk of relapse or progression (AETHERA): a randomised, double-blind, placebo-controlled, phase 3 trial.. *Lancet (London, England)*.

[6] National Cancer Institute. (2010). Common Terminology Criteria for Adverse Events (CTCAE) version 4.0.. https://evs.nci.nih.gov/ftp1/CTCAE/About.html.

[7] Senter Peter D, Sievers Eric L (2012). The discovery and development of brentuximab vedotin for use in relapsed Hodgkin lymphoma and systemic anaplastic large cell lymphoma.. *Nature biotechnology*.

[8] Singh A, Singh D, Bhoria U (2014). Infusion reactions associated with the use of biologic medicines in cancer therapy.. *Oncocytology*.

[9] Younes Anas, Bartlett Nancy L, Leonard John P, Kennedy Dana A, Lynch Carmel M, Sievers Eric L, Forero-Torres Andres (2010). Brentuximab vedotin (SGN-35) for relapsed CD30-positive lymphomas.. *The New England journal of medicine*.

